# Lipid-lowering and antioxidant effects of *Polygonatum* fermented liquor: a study on intestinal microbiota and brain–gut axis in mice

**DOI:** 10.3389/fnut.2024.1428228

**Published:** 2024-08-16

**Authors:** Xuan Yang, Leyao Fang, Junxi Shen, Zhoujin Tan, Wenhong Zeng, Maijiao Peng, Nenqun Xiao

**Affiliations:** ^1^School of Pharmacy, Hunan University of Chinese Medicine, Changsha, Hunan, China; ^2^Hunan University of Chinese Medicine, Changsha, Hunan, China; ^3^Xinhua County Chiyou Distillery, Xinhua, Hunan, China

**Keywords:** *Polygonatum* fermented liquor, lipid-lowering, antioxidant, gut microbiota, brain–gut axis

## Abstract

**Introduction:**

This study aims to investigate the effects of *Polygonatum* fermented liquor (PFL) on improving lipid metabolism and oxidative stress in mice by regulating the gut microbiota.

**Methods:**

Forty SPF-grade male Kunming mice were randomly divided into four groups: normal control group (NC), general liquor group (GC), fresh *Polygonatum* fermented liquor group (FPC), and nine-steam-nine-bask *Polygonatum* fermented liquor group (NPC). Each group was administered with sterile water, general liquor, fresh *Polygonatum* fermented liquor, and nine-steam-nine-bask *Polygonatum* fermented liquor, respectively, by gavage. The mice's liver, brain tissue, serum, and intestinal contents were collected. The indicators of oxidative stress in the liver, four blood lipid indicators, gamma-aminobutyric acid (GABA), and brain-derived neurotrophic factor (BDNF) levels in the brain tissue were measured, liver hematoxylin and eosin (HE) staining was performed, and the gut microbiota in the small intestine were analyzed using 16S rRNA second-generation sequencing technology.

**Results:**

Compared with the NC group, the NPC group showed significantly increased liver glutathione peroxidase (GSH-Px) content in mice (*p* < 0.05), reduced number of lipid droplets in the liver cells, and increased GABA and BDNF content in the brain tissues. The NPC group regulated lipid metabolism by lowering low-density lipoprotein cholesterol (LDL-C) and increasing high-density lipoprotein cholesterol (HDL-C) content in the mouse serum. Gut microbiota analysis showed significant changes in the gut microbiota of mice in the FPC and NPC groups, with increased richness and species diversity. These two groups increased the abundance of beneficial bacteria such as *Lactobacillus*, unclassified *Muribaculaceae*, unclassified *Bacilli*, and uncultured *Bacteroidales bacterium* while reducing the abundance of harmful bacteria such as *Candidatus Arthromitus*, and *Staphylococcus*, with a particularly significant reduction in *Staphylococcus* (*p* < 0.05). It is speculated that the two types of PFL may exert lipid-lowering and antioxidant effects by modulating the abundance of these dominant bacteria. Further studies showed that various environmental factors are closely related to the dominant gut bacteria. Malondialdehyde (MDA) was significantly negatively correlated with *Lactobacillus* and unclassified *Bacilli*, superoxide dismutase (SOD) was significantly negatively correlated with *Staphylococcus* (*p* < 0.01) and significantly negatively correlated with *Candidatus Arthromitus* (*p* < 0.05), and HDL-C was significantly negatively correlated with *Staphylococcus* and *Facklamia* (*p* < 0.05).

**Discussion:**

The two types of PFL chosen in this study may exert lipid-lowering and antioxidant effects by modulating the composition and function of the gut microbiota, providing guidance for the industrial application of *Polygonatum*.

## 1 Introduction

*Polygonatum sibiricum* (PS), known as Huang Jing, is a dry rhizome of *Polygonatum* Redouté, *Polygonatum kingianum* Collett & Hemsl, or *Polygonatum cyrtonema* Hua^1^. Its main components include *Polygonatum* polysaccharides, *Polygonatum* oligosaccharides, *Polygonatum* saponins, alkaloids, and flavonoids (1, 2). *Polygonatum* is well-known as a traditional medicinal herb and functional food in China, which has been used as an anti-aging, anti-inflammatory, anti-osteoporotic agent, as well as an immunity booster and sleep enhancer (3–7). Studies have shown that *Polygonatum* can enhance immunity and promote health by regulating the gut microbiota (8, 9). However, fresh *Polygonatum* is highly irritating and may cause diarrhea in individuals with spleen and stomach deficiency (10, 11). Recent research (12) has shown that processing *Polygonatum* using the nine-steam-nine-bask method can significantly improve its hypoglycemic and antioxidant activities *in vitro* and reduce its irritability. Yellow liquor is a typical low-alcohol grain-fermented beverage (generally 14% vol) containing various bioactive substances such as amino acids, peptides, polysaccharides, oligosaccharides, and phenolics (13). PFL is yellow liquor made by fermenting fresh *Polygonatum* powder or nine-steam-nine-bask *Polygonatum* powder, a characteristic medicinal and edible material from Xinhua, Hunan, with glutinous and other auxiliary materials. Despite the known benefits of *Polygonatum*, there is limited research on its effects when fermented and its impact on the brain–bacteria–gut axis in a murine model.

Gut microbiota is crucial to human health. Based on their different physiological functions, gut microbiota can be classified into beneficial, neutral, and harmful bacteria. Gut microbiota maintains a dynamic balance, and its deficiency or disruption can lead to metabolic diseases (14). Oxidative stress is a major cause of decreased immunity. Gut microbiota regulates oxidative stress in the liver and brain (15). Oxidative stress of the liver increases the risk of hepatocyte death, leading to various liver diseases (16). Studies have shown that gut microbiota protects hepatocytes and alleviates liver oxidative stress by regulating oxidative stress indicators such as malondialdehyde (MDA), superoxide dismutase (SOD), and glutathione peroxidase (GSH-Px) (17–19). Oxidative stress in the brain can damage neuron integrity, leading to cell death or various mental disorders (20, 21). Brain-derived neurotrophic factor (BDNF) is a key neurotrophic protein, and gamma-aminobutyric acid (GABA) is an important inhibitory neurotransmitter that protects neurons from oxidative damage. Studies have shown that probiotics can promote BDNF production and convert glutamate to GABA, thereby alleviating brain oxidative stress (22, 23). Our previous research (24) indicated that moderate alcohol consumption can regulate the structure of gut microbiota and digestive enzyme activity in mice, thus positively impacting health. Therefore, this study compares the effects of FPFL and NPFL on the gut microbiota of mice and examines the liver and brain oxidative stress indicators to explore the impact of PFL on oxidative stress levels in mice.

Gut microbiota can regulate blood lipids. The main components of blood lipids are triglycerides and cholesterol. Triglycerides are involved in energy metabolism, while cholesterol is used to synthesize cell membranes, steroid hormones, and bile acids. Gut microbiota can produce cholesterol oxidase to degrade cholesterol, thus regulating cholesterol levels (25). Additionally, short-chain fatty acids, the metabolic products of gut microbiota, can inhibit liver fatty acid synthase, reducing blood lipid levels (26). Sugar intake is closely related to blood lipids. Excessive sugar intake can be converted into large amounts of triglycerides, leading to elevated blood lipid levels (27). The total sugar content of general liquor exceeds 100 g/L and therefore it is classified as a sweet yellow liquor. This study compares the effects of FPFL and NPFL on gut microbiota and measures four blood lipid indicators to explore the impact of PFL on blood lipid levels in mice.

## 2 Materials and methods

### 2.1 Animals and housing

Studies have shown that there are sex differences in alcohol tolerance in Kunming mice, and male mice are more sensitive to alcohol (28). Due to the larger proportion of alcohol consumption among men in the general population and support from literature for this consumption pattern, the use of male mice to simulate human alcohol consumption is more representative. Forty SPF-grade Kunming mice, weighing 18–22 g, were purchased from Hunan Slack Jingda Experimental Animal Co., Ltd. (Hunan, China), with the animal license number SCXK (Xiang) 2019-0004. The mice were housed in separate cages in a barrier environment at the Experimental Animal Center of Hunan University of Chinese Medicine, with a room temperature of 23–25°C, relative humidity of 50–70%, and a 12-h light–dark cycle, with free access to food and water. The feed was provided by the Experimental Animal Center of Hunan University of Chinese Medicine. All experiments and procedures involving animals were conducted by the protocols approved by the Ethics Committee of Experimental Animals of Hunan University of Chinese Medicine, with the ethics approval number LL2023032901.

### 2.2 *Polygonatum* fermented liquor

General liquor (GL), fresh *Polygonatum* fermented liquor (FPFL), and nine-steam-nine-bask *Polygonatum* fermented liquor (NPFL) were provided by Chiyou Distillery in Xinhua County (Loudi City, China). The FPFL and NPFL were prepared according to the patent “A and Its Preparation Method and Application” (application number: 2023110416934). The fresh *Polygonatum* and nine-steam-nine-bask *Polygonatum* used for fermentation were sourced from Xinhua County and were inspected by Licheng Testing and Certification Group Co., Ltd. The specific steps followed for this experiment are as follows.

The glutinous rice is mixed with 1.96% of fresh *Polygonatum* powder or nine-steam-nine-bask *Polygonatum* powder, soaked, steamed until cooked, and then cooled to 28°C as the embryo. After blending 0.25% fermentation coke with 99.75% germ material, the mixture is poured into a ceramic jar at 28°C for saccharification and fermentation for 6 days. It is then transferred to another ceramic jar and fermented at 28°C for an additional 6 days based on the mass volume ratio of raw materials and cold boiled water of 1 kg:0.8 L. The resulting mixture undergoes press filtration, sterilization, clarification, and adjustment of the sugar degree to 132 g/L and the alcohol level to 11.6%.

### 2.3 Animal grouping and intervention

Previous research of our research group had demonstrated that a 10% alcohol aqueous solution, when used as the daily drinking water for mice, can modulate the composition of intestinal flora and the relative activity of digestive enzymes, exerting a positive impact on the human body. After a 3-day acclimation period, 40 male Kunming mice were randomly divided into four groups: the NC, GC, FPC, and NPC groups, with 10 mice in each group, housed separately. The GC, FPC, and NPC groups were given intragastric administration of GL, FPFL, and NPFL, respectively, at 0.35 mL per mouse, twice daily, for six consecutive weeks. The NC group received sterile water intragastrically with the same volume, frequency, and duration as the other groups.

### 2.4 General observation of animals

During the 6-week continuous intragastric administration, the mice had free access to food and water. Weekly observations and recordings of general behavioral changes (body weight, food intake, water intake, fecal characteristics, mental state, activity, and physical signs) were made.

### 2.5 Detection of oxidative stress levels in the liver

After the mice were quickly killed by cervical dislocation on a sterile operation platform, the liver was taken out, and two sets of 0.1 g of liver tissues were weighed. One mL of MDA, 1 mL of SOD, and 1 mL of GSH-Px extract were added to the liver tissues separately in an ice bath for homogenization. Then, they were centrifuged at 2,500 r/min at 4°C for 10 min. The supernatant was used to measure MDA, SOD, and GSH-Px levels, strictly following the instructions in the enzyme-linked immunosorbent assay (ELISA) kits.

### 2.6 Liver HE staining

Paraffin sections were dewaxed with xylene, rehydrated through a graded ethanol series to water; stained with hematoxylin for 25 min followed by water washing, differentiated with hydrochloric acid ethanol for 30 s, soaked in water for 15 min, stained with 0.5% eosin for 2 min, washed with water; dehydrated conventionally with ethanol, xylene carbolic acid, and xylene, and sealed with neutral resin. The pathological morphology of the liver tissues was observed using an optical microscope and CaseViewer software.

### 2.7 Detection of GABA and BDNF content in the brain tissue

Five mice were randomly selected from each group, and brain samples were placed at −20°C after dissection. The frozen brain tissue was accurately weighed and nine times the volume of physiological saline was added. The tissue was homogenized in an ice-water bath to make a 10% liver homogenate and centrifuged at high speed (4°C, 3,000 r/min) for 20 min. The supernatant was used for the determination of GABA and BDNF content (in the serum) using enzyme-linked immunosorbent assay (ELISA).

### 2.8 Detection of four blood lipid levels

After 12 h of fasting and water deprivation, blood was collected from the mice by enucleation under sterile conditions. The blood samples were left to stand at 4°C for 1–2 h. The supernatant was then transferred to centrifuge tubes and centrifuged at 3,000 r/min for 15 min in a high-speed centrifuge. The serum was used to measure four blood lipid levels: TC, TG, LDL-C, and HDL-C, strictly following the instructions provided with the ELISA kits.

### 2.9 16S rRNA gene high-throughput sequencing

**① Sample collection:** The mice were euthanized by cervical dislocation and immediately placed on a clean bench. A section of the small intestine was taken as a test sample. The intestinal contents were squeezed out with sterilized tweezers, placed individually in 1.5 mL sterilized Eppendorf tubes, labeled, quickly frozen in liquid nitrogen, and then stored at −80°C (29, 30). All samples were sent to Biomarker Technologies Corporation (Beijing, China) for 16S rRNA gene high-throughput sequencing.

**② Species notes:** The raw reads obtained from sequencing were filtered using Trimmomatic v0.33 software; primer sequences were identified and removed using Cutadapt 1.9.1 software to obtain clean reads without primer sequences. The DADA2 method in QIIME2 2020.6 was used for denoising, paired-end sequence merging, and chimera sequence removal to obtain amplicon sequence variants (ASVs). The ASV feature sequences were compared with reference sequences in the SILVA database to obtain classification information for each ASV. Using QIIME2 software, the sequence number of each sample in the ASV abundance matrix was randomly extracted at different depths, and the sequence number extracted at each depth and the corresponding ASV number were used to draw rarefaction curves. Venn diagrams were used to show the number of shared and unique ASVs between groups.

**③ Alpha diversity analysis:** The Chao1 index, observed species index, Shannon index, and Simpson index of each group were calculated to compare the richness and evenness of ASVs between different samples.

**④ Beta diversity analysis:** The Bray–Curtis distance was used to analyze the changes in microbial community structure between the samples. Principal coordinate analysis (PCoA) and non-metric multidimensional scaling (NMDS) were used for visualization.

**⑤ Characteristic microbiota analysis:** QIIME2 software was used to obtain the composition and abundance tables of each sample at different taxonomic levels, which were presented as bar charts. Linear discriminant analysis effect size (LEfSe) was used with default parameters to detect differential abundance among different taxa. The random forest analysis of samples from different groups was performed using QIIME2′s default settings.

**⑥ Correlation analysis:** The Spearman correlation coefficient among characteristic bacteria was calculated. The correlation network was constructed by Cytoscape 3.7.2 software to explore the synergistic/competitive relationship among characteristic bacteria. Redundancy analysis (RDA) was used to investigate the interactions between the characteristic flora of intestinal contents and environmental factors.

**⑦ Functional predictive analysis:** PICRUSt2 was used to predict the functional abundance of samples in the KEGG database and to perform LEfSe analysis to identify the metabolic pathways with different abundances between groups. Spearman analysis was used to explore the correlation between blood lipids and oxidative indices with the gut microbiota in the small intestine.

### 2.10 Statistical analysis

Data were analyzed with IBM SPSS (v 25.0) software, and when the data conformed to the normal distribution and variance uniformity, one-way analysis of variance (ANOVA) test was used to compare the differences among multiple groups. The least significant difference (LSD) method was used for pairwise comparison between groups. When the data did not conform to the normal distribution or variance was inconsistent, the Kruskal–Wallis test in non-parametric test and Bonferroni correction were used. A *P*-value < 0.05 was considered statistically significant.

## 3 Results

### 3.1 General physical and fecal characteristics of mice

Mice in the NC group exhibited good mental state, high activity levels, smooth and shiny fur, and clean, dry perianal and tail areas. Their feces were brown, spindle-shaped, and moderately firm. Mice in the GC group showed reduced activity and preferred to curl up, huddle together, and squint. Their fur lost its shine, and their feces were smaller and harder. Mice in the FPC group exhibited good mental state, were more active than those in the NC group, had shinier fur, and their feces were spindle-shaped and moderately firm. Mice in the NPC group exhibited good mental states, were more active than those in the NC group, had shinier fur, and their feces were spindle-shaped and moderately firm.

### 3.2 Effects of PFL on mouse body weight

**Figure 1:**
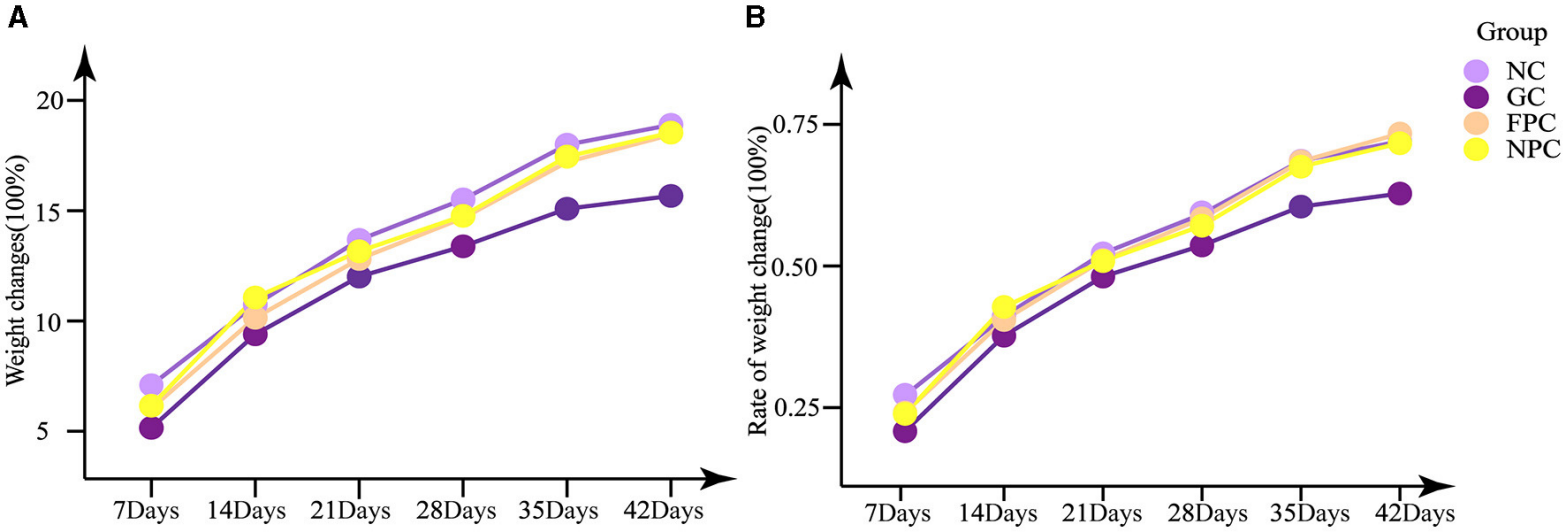
Effects of PFL on weight change and weight change rate of mice. **(A)** Weight changes; **(B)** rate of weight change. NC, normal control group; GC, general liquor group; FPC, fresh *Polygonatum* fermented liquor group; NPC, nine-steam-nine-bask *Polygonatum* fermented liquor group.

As shown in Figures 1A, B, the GC, FPC, and NPC groups exhibited varying degrees of weight loss, but the differences in body weight changes were not statistically significant (*p* > 0.05), nor were the differences in the rate of weight change (*p* > 0.05). Among them, the GC group had smaller weight changes and rates of change compared to the NC group, while the FPC and NPC groups had weight changes and rates of change close to those of the NC group.

### 3.3 Effects of PFL on hepatic oxidative stress in mice

**Figure 2:**
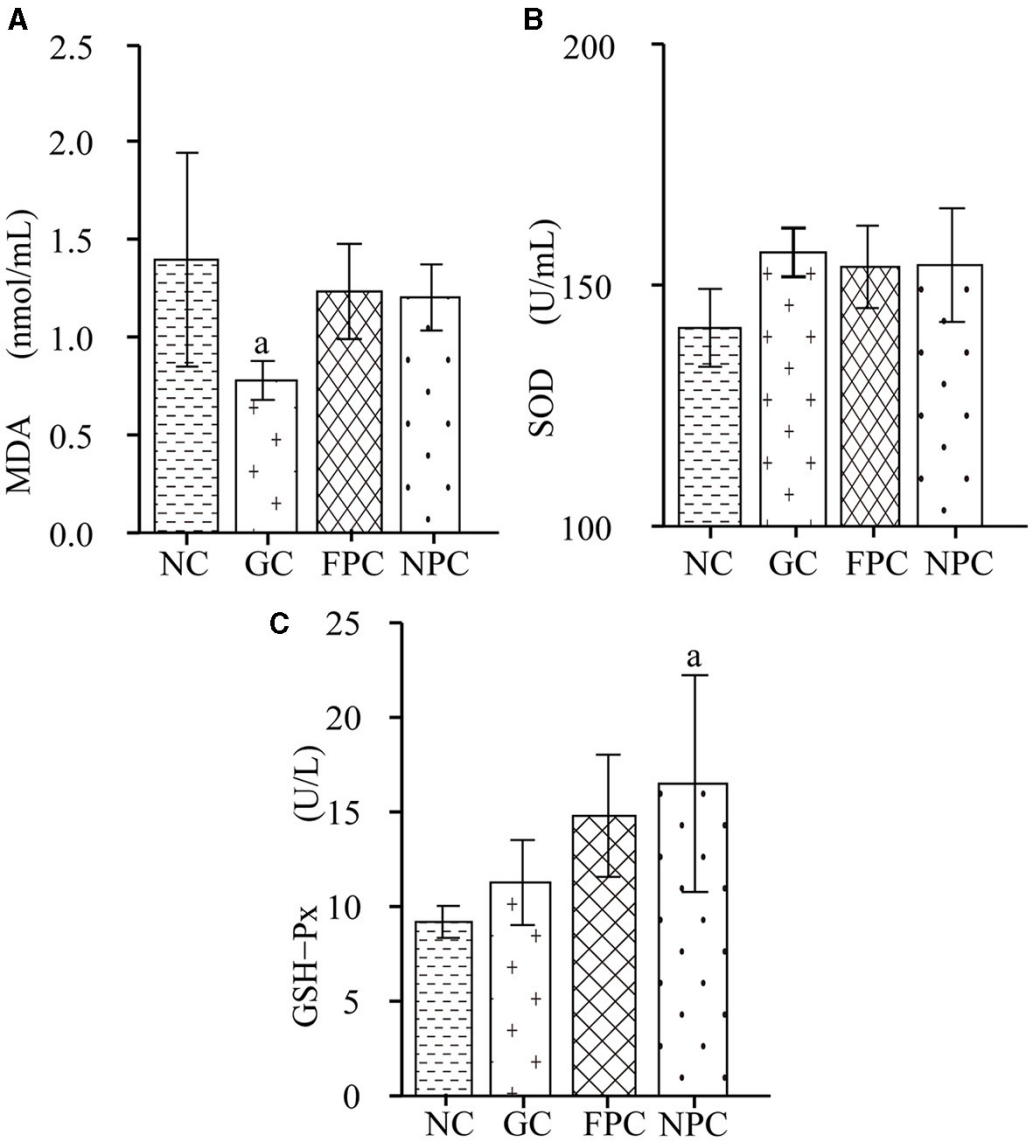
The impact of PFL on the levels of BDNF and GABA in the mouse brain. **(A)** MDA levels; **(B)** SOD levels; **(C)** GSH-Px levels. NC, normal control group; GC, general liquor group; FPC, fresh *Polygonatum* fermented liquor group; NPC, nine-steam-nine-bask *Polygonatum* fermented liquor group. a, The difference is evident when compared with the NC group.

Superoxide dismutase (SOD) and glutathione peroxidase (GSH-Px) are essential components of the body's antioxidant system. SOD catalyzes the conversion of superoxide anion radicals into oxygen and hydrogen peroxide, playing a crucial role in maintaining the balance between oxidation and antioxidation in the body. Its levels reflect the body's ability to remove oxygen-free radicals and are closely related to the occurrence and development of many diseases. GSH-Px catalyzes the reaction of reduced glutathione with reactive oxygen species to form oxidized glutathione, protecting biological membranes from oxidative damage and maintaining normal cellular function. It can protect the liver, enhance the body's immunity, counteract the damage caused by harmful metal ions, and increase the body's resistance to radiation. Free radicals act on lipids to produce peroxidation reactions, with malondialdehyde (MDA) as the final product, which can cause cross-linking and polymerization of macromolecules such as proteins and nucleic acids, leading to cytotoxicity. When lipid peroxidation in the body increases and excessive oxygen free radicals are generated, the levels of antioxidant enzymes such as SOD and GSH-Px in the body increase, thereby removing excess oxygen free radicals and maintaining the dynamic balance between oxidation and antioxidation. As shown in Figure 2A, compared with the NC group, the MDA levels in all liquor groups decreased, with the GC group having a significantly lower MDA level than the NC group (*p* = 0.038, *p* < 0.05). As shown in Figure 2B, compared with the NC group, the SOD levels in mice from all liquor groups increased, but the differences were not significant (*p* > 0.05). As shown in Figure 2C, compared with the NC group, the GSH-Px levels in all liquor groups were significantly higher, with the NPC group being significantly higher than the NC group (*p* = 0.022, *p* < 0.05). After intragastric administration, the antioxidant capacity of the liver in mice was the strongest in the NPC group, followed by the FPC group.

### 3.4 Effects of PFL on liver HE staining in mice

**Figure 3:**
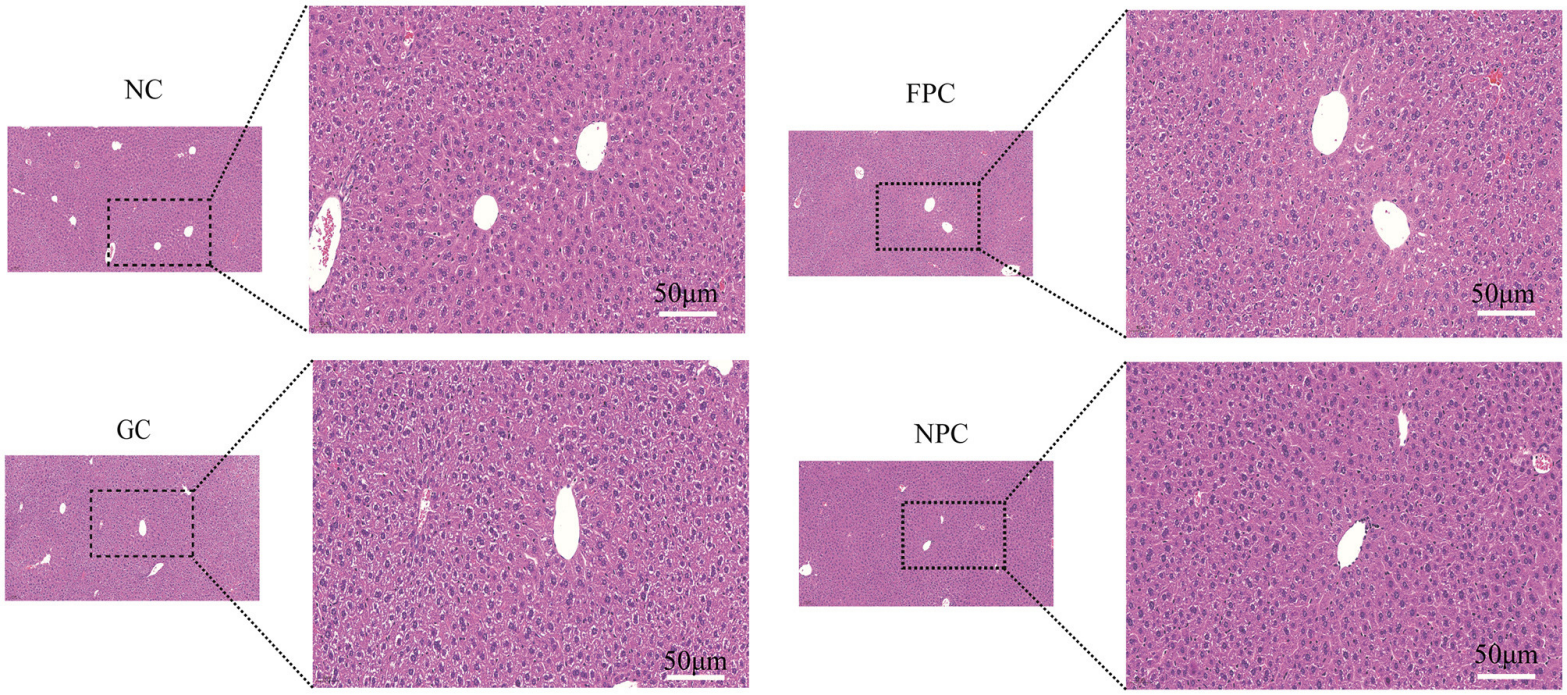
The impact of PFL on HE staining of mouse liver. NC, normal control group; GC, general liquor group; FPC, fresh *Polygonatum* fermented liquor group; NPC, nine-steam-nine-bask *Polygonatum* fermented liquor group.

Hematoxylin and eosin (HE) staining is a widely used method in medical diagnosis. Hematoxylin is a basic dye that stains chromatin in the cell nucleus and nucleic acids in the cytoplasm with a purplish-blue color. Eosin is an acidic dye that stains components in the cytoplasm and extracellular matrix red, thus distinguishing various cell types and histological features. As shown in Figure 3, HE staining results showed that the hepatic lobule structure in the NC group of mice was intact, with neatly arranged liver cells, clear structure, uniform cytoplasm, a small number of lipid droplets in the cells, and no fatty degeneration. In the NPC group, the hepatic lobule structure was intact, liver cells were neatly arranged with a clear structure and uniform cytoplasm, and the number of lipid droplets in the cells significantly reduced, with no fatty degeneration. Therefore, the NPC group shows the most effective reduction in liver fat accumulation.

### 3.5 Effects of on BDNF and GABA levels in mice brains

**Figure 4:**
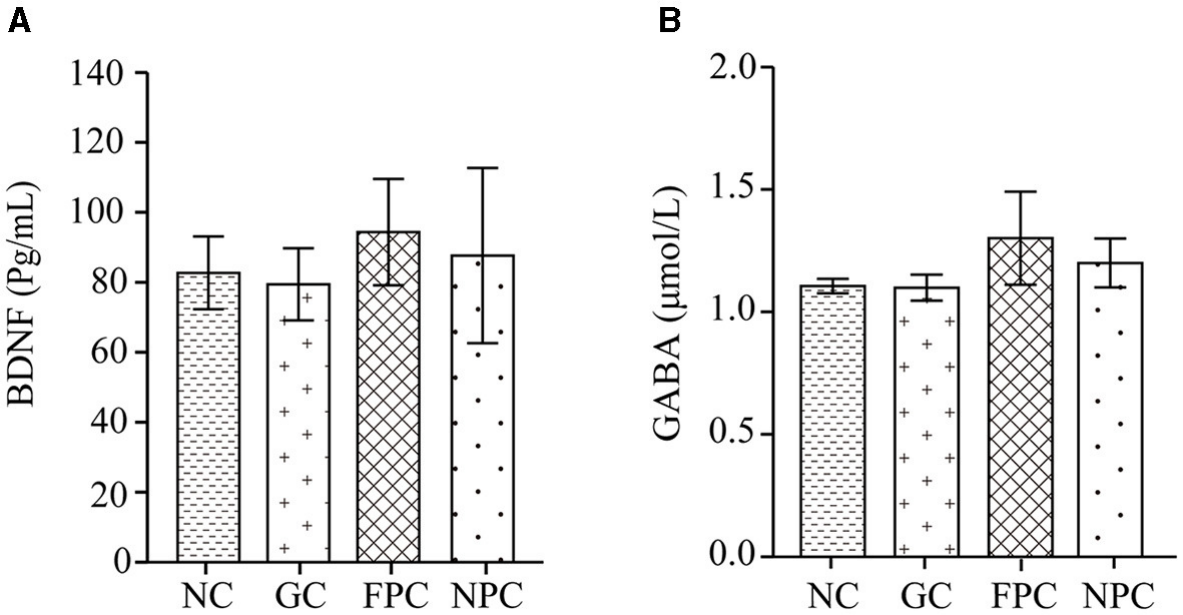
The impact of PFL on the levels of BDNF and GABA in the mouse brain. **(A)** BDNF levels; **(B)** GABA levels. NC, normal control group; GC, general liquor group; FPC, fresh *Polygonatum* fermented liquor group; NPC, nine-steam-nine-bask *Polygonatum* fermented liquor group.

BDNF plays a crucial role in neuron survival, differentiation, and synaptic plasticity, influencing learning and memory abilities and has potential therapeutic effects on cognitive changes related to neurodegenerative diseases. GABA is an important inhibitory neurotransmitter in the central nervous system. It reduces neuronal activity, prevents overheating of nerve cells, calms the nervous system, and counteracts anxiety. GABA and BDNF not only maintain neuronal function but also improve hippocampal neurogenesis. They regulate each other; GABA promotes the transport and expression of BDNF, and BDNF enhances the transcriptional activity of GABA. Experimental results in Figures 4A, B show that compared with the NC group, the levels of BDNF and GABA in the FPC and NPC groups were increased, but the differences were not significant (*p* > 0.05). The BDNF and GABA levels in different groups of mice brains were in the following order: GC < NC < NPC < FPC. This suggests that FPFL and NPFL may reduce the harmful effects of alcohol on the brain. The protective effect of the FPC group on the mouse hippocampus was the best, followed by the NPC group.

### 3.6 Effects of PFL on the four blood lipid indices in mice

**Figure 5:**
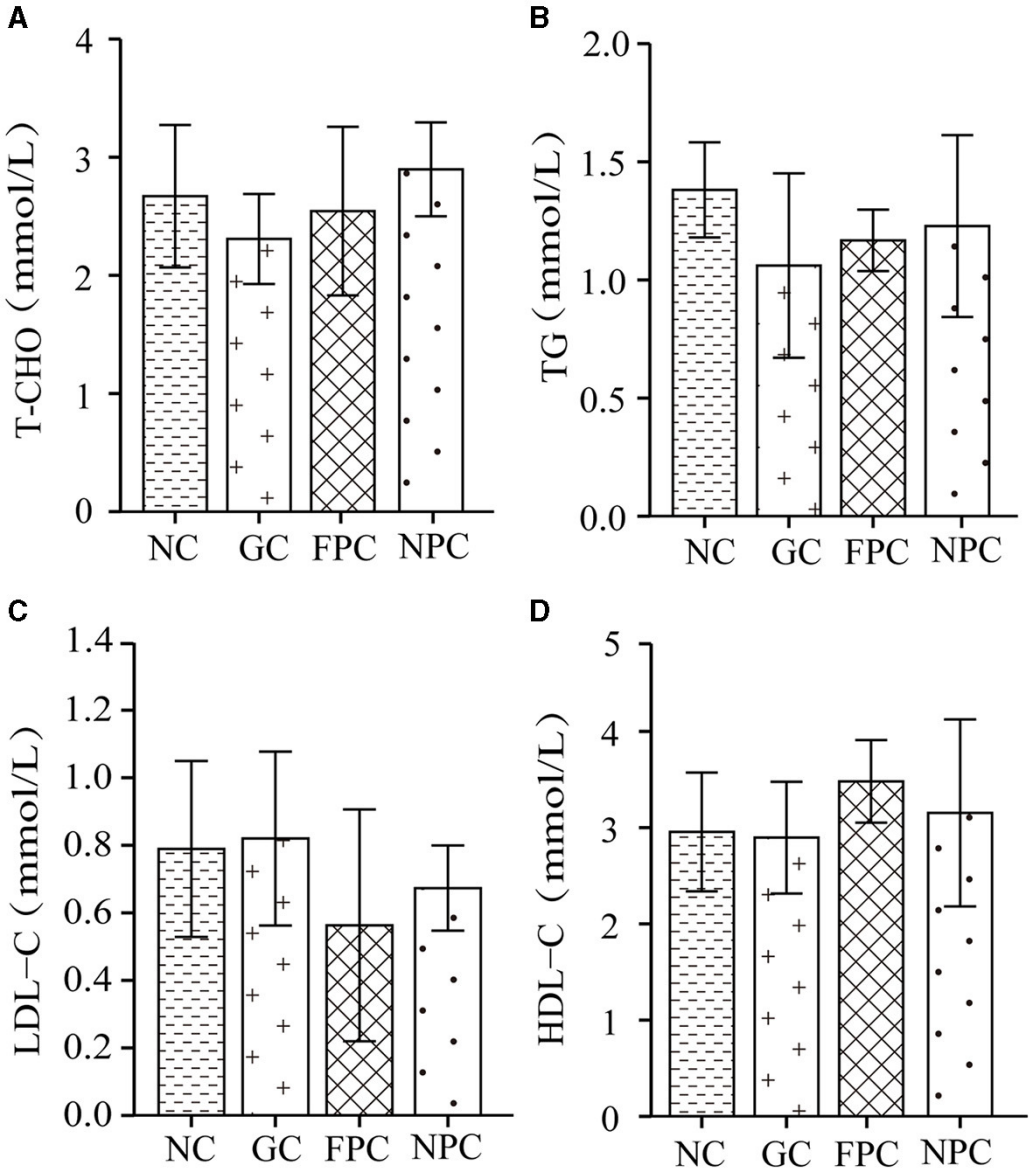
The impact of PFL on the four indicators of blood lipids in mice. **(A)** T-CHO levels; **(B)** TG levels; **(C)** LDL-C levels; **(D)** HDL-C levels. NC, normal control group; GC, general liquor group; FPC, fresh *Polygonatum* fermented liquor group; NPC, nine-steam-nine-bask *Polygonatum* fermented liquor group.

The four blood lipid indices, commonly used in clinical examinations for lipid abnormalities, include triglycerides (TG), total cholesterol (T-CHO), high-density lipoprotein cholesterol (HDL-C), and low-density lipoprotein cholesterol (LDL-C). Measuring serum lipid levels can be used to evaluate the fat metabolism level in mice. As shown in Figure 5A, compared with the NC, the T-CHO levels in the FPC and NPC groups were reduced, but the differences were not significant (*p* > 0.05). The order of T-CHO levels in the serum of different groups of mice was as follows: NPC < FPC < NC < GC. As shown in Figure 5B, compared with the NC group, the TG levels in the FPC and NPC groups were reduced, but the differences were not significant (*p* > 0.05). The order of TG levels in the serum of different groups of mice was as follows: NPC < FPC < NC < GC. As shown in Figure 5C, compared with the NC, LDL-C levels decreased in the FPC and NPC groups, while they increased in the GC. The order of LDL-C levels in the serum of different groups of mice was as follows: NPC < FPC < NC < GC. As shown in Figure 5D, compared with the NC group, HDL-C levels increased in the FPC and NPC groups. The order of HDL-C levels in the serum of different groups of mice was as follows: NC < GC < FPC < NPC. Therefore, the NPC group displayed the best lipid-lowering effect in mice, followed by the FPC group.

### 3.7 Effects of PFL on the intestinal microbiota of mice

**Figure 6:**
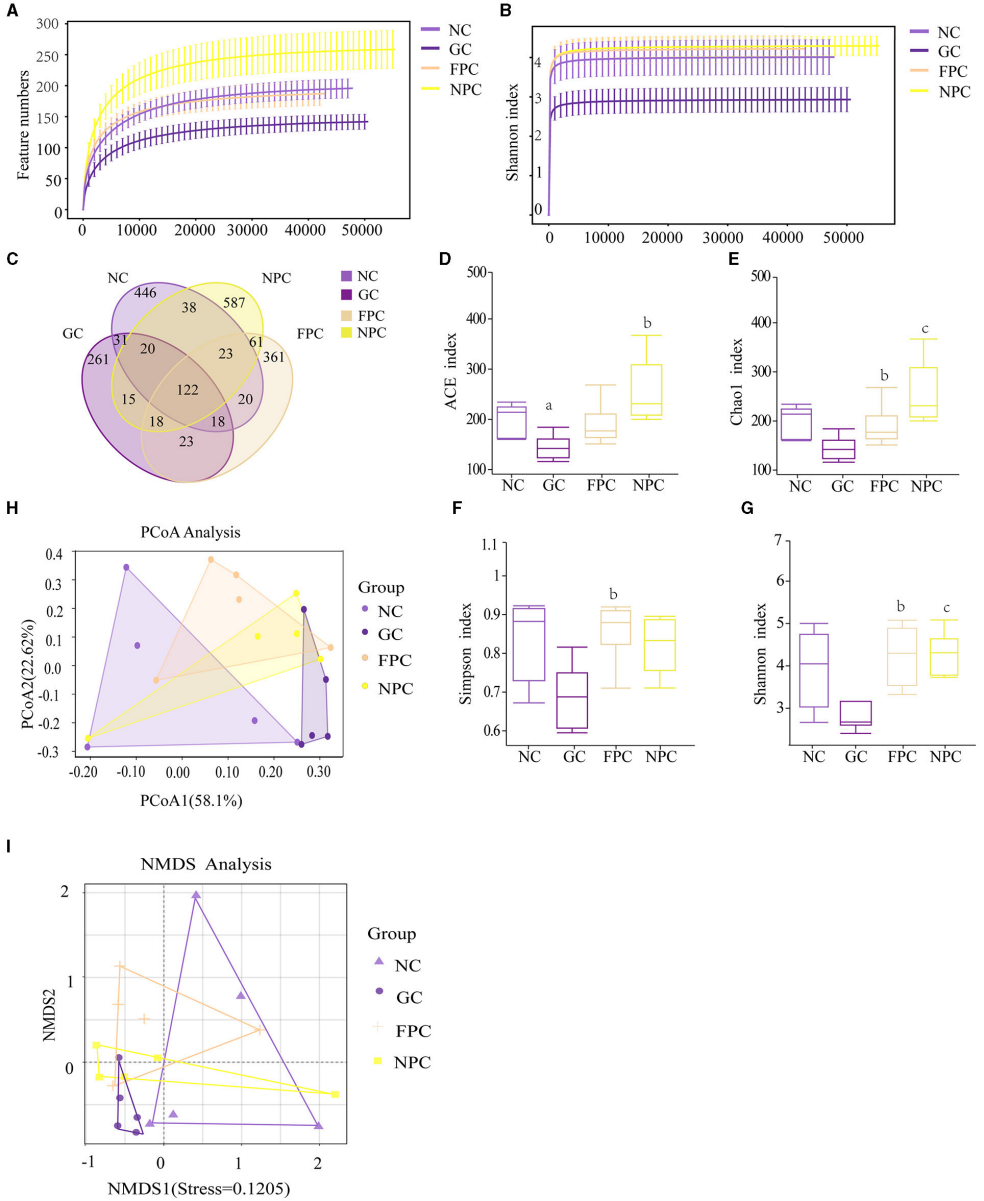
The impact of PFL on the intestinal microbiota of mice. **(A)** Feature numbers; **(B)** Shannon index; **(C)** ASV quantity; **(D)** ACE index; **(E)** Chao1 index; **(F)** Simpson index; **(G)** Shannon index; **(H)** pCoA analysis; **(I)** NMDS analysis. NC, normal control group; GC, general liquor group; FPC, fresh *Polygonatum* fermented liquor group; NPC, nine-steam-nine-bask *Polygonatum* fermented liquor group. a, The difference is evident when compared with the NC group; b, The difference is evident when compared with the GC group; c, The difference is evident when compared with the FPC group.

Rarefaction curves are used to verify whether the sequencing data volume is sufficient to reflect the species diversity in the samples and indirectly indicate the species richness in the samples. Within a certain range, if the curve rises sharply with the increase in sequencing reads, it indicates that a large number of species have been discovered in the community; if the curve levels off, it indicates that the number of species in this environment will not significantly increase with more sequencing reads. As shown in Figures 6A, B, with the increase in sample size, the total number of ASVs no longer increases significantly, and the curve levels off, indicating that the sample size in this study is sufficient to reflect the species composition of the community.

As shown in Figure 6C, the NC group had 718 ASVs, with 446 unique ASVs; the GC group had 508 ASVs, with 261 unique ASVs; the FPC had 646 ASVs, with 361 unique ASVs; and the NPC group had 884 ASVs, with 587 unique ASVs. The four groups shared a total of 2,044 ASVs. The results showed that the total number of ASVs decreased in the GC group, with a corresponding reduction in unique ASVs. There was no significant change in the total number of ASVs in the FPC group; however, both the total number and the number of unique ASVs increased in the NPC group. This indicates that the intake of different liquors significantly altered the species present and diversity of the intestinal microbiota in mice.

As shown in Figures 6D–G, alpha diversity analysis of microbial communities was used to compare species richness (ACE index, Chao1 index) and diversity (Simpson index, Shannon index) among the four groups. As shown in the figures, the ACE and Chao1 indices were ordered from low to high as follows: GC < NC < FPC < NPC (NCvsGC, *p* = 0.024, *p* < 0.05;GCvsNPC, *p* = 0.008, *p* < 0.01). The Simpson index was ordered from low to high as follows: GC < NPC < NC < FPC (GCvsFPC, *p* = 0.016, *p* < 0.05). It can be seen that the NPC group can effectively increase the species richness of the microbiota in mice.

As shown in Figures 6H, I, beta diversity was assessed using the Bray–Curtis distance algorithm, and significant differences in microbial community composition were found between different (*P* = 0.019), as confirmed by analysis of similarity (ANOSIM) analysis. The closer the distance between the two on the coordinate axis, the more similar their community composition in the respective dimensions. PCoA analysis showed that the contribution rate of the horizontal axis PCoA1 was 58.1%, and the contribution rate of the vertical axis PCoA2 was 17.2%. The NC samples partially overlapped with the FPC and NPC samples but did not overlap with the GC. Compared to the NC, the community structure of the intestinal microbiota in the FPC and NPC changed significantly but showed some similarity, whereas the community structure in the GC was significantly different from that in the NC. The results of the NMDS analysis were consistent with the PCoA analysis, indicating that FPFL and NPFL affect body functions by altering the intestinal microbiota.

### 3.8 Effects of PFL on the species composition of intestinal microbiota in mice

**Figure 7:**
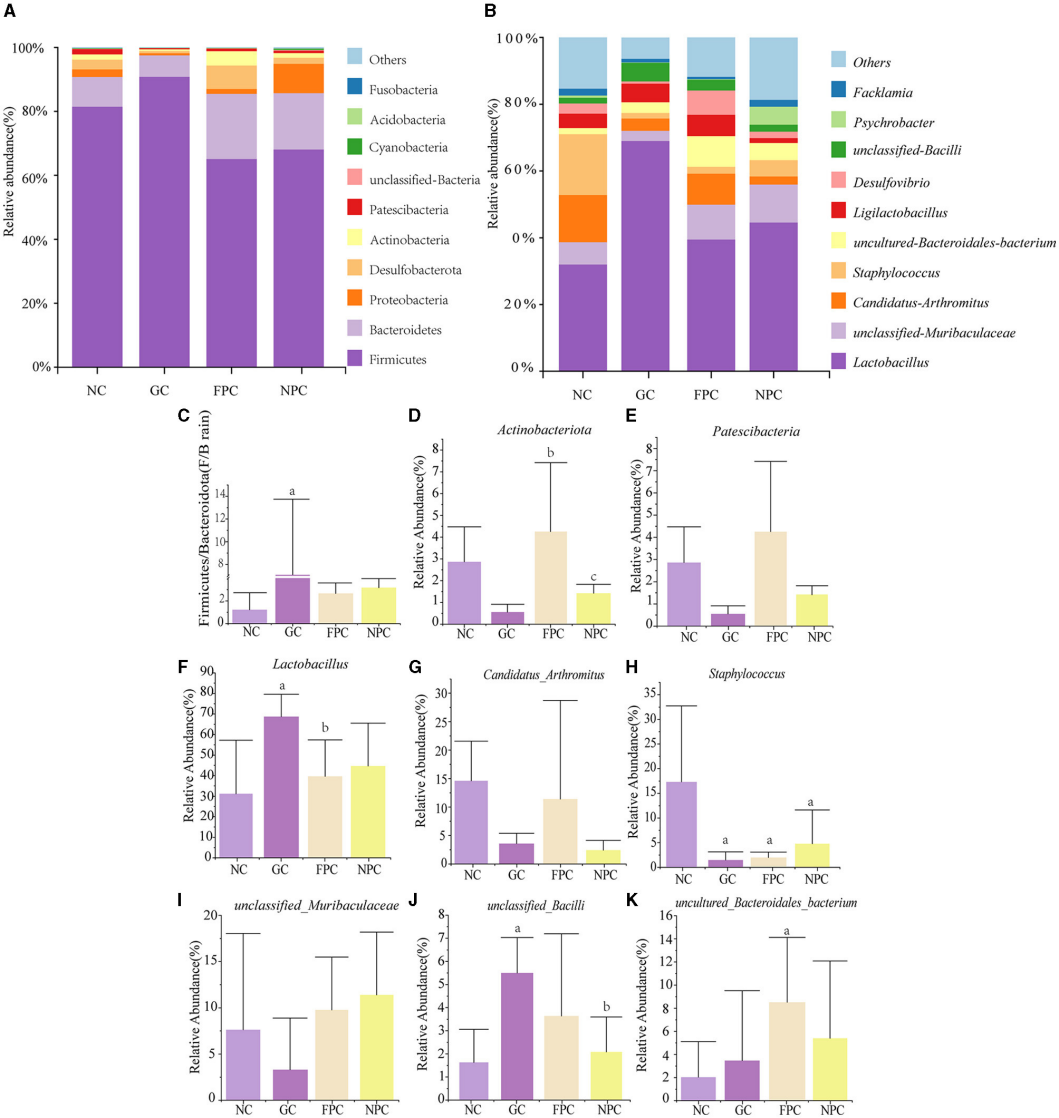
Impact of PFL on the species composition of mouse intestinal microbiota. **(A)** Phylum-level intestinal microbiota composition; **(B)** genus-level intestinal microbiota composition; **(C)** F/B ratio; **(D, E)** dominant phyla in the contents of all groups; **(F–K)** dominant genus in the intestinal microbiota contents of all the groups. NC, normal control group; GC, general liquor group; FPC, fresh *Polygonatum* fermented liquor group; NPC, nine-steam-nine-bask *Polygonatum* fermented liquor group. a, The difference is evident when compared with the NC group; b, The difference is evident when compared with the GC group; c, The difference is evident when compared with the FPC group.

To investigate the effect of PFL on the intestinal microbiota of mice, we selected the top 10 dominant phyla and genera that have a relative abundance of more than 1% and used bar charts to represent their abundance (Figures 7A, B). At the phylum level, Firmicutes, Bacteroidota, Proteobacteria, and Desulfobacterota were the predominant phyla with relatively high proportions in the four groups (Figure 7A). At the genus level, *Lactobacillus*, unclassified *Muribaculaceae, Candidatus Arthromitus*, and *Staphylococcus* were the dominant genera with relatively large proportions in the four groups (Figure 7B). There were significant changes at the phylum level in each liquor group compared to the NC group. Figure 7C shows the abundance ratio of Firmicutes to Bacteroidota (F/B ratio). Compared to the NC group, the F/B ratio significantly increased in the GC group (*p* = 0.016, *p* < 0.05), while it was significantly reduced in the FPC and NPC groups compared to the GC group. Figures 7D, E show that compared to the NC group, the GC group significantly decreased the abundance of *Actinobacteriota* and *Patescibacteria*, but this trend was reversed in the FPC and NPC groups. As shown in Figures 7F–K, further observation at the genus level revealed that the FPC and NPC groups upregulated the abundance of beneficial bacteria such as *Lactobacillus*, unclassified Muribaculaceae, unclassified Bacilli, and uncultured Bacteroidales bacterium while downregulating harmful genera such as *Candidatus Arthromitus* and *Staphylococcus*, with *Staphylococcus* showing a significant decrease (*p* = 0.011, *p* < 0.05; *p* = 0.033, *p* < 0.05). This suggests that FPFL and NPFL may promote organism health by adjusting the ratio of dominant phyla and genera in the intestine.

### 3.9 Effect of PFL on the characteristic microbiota of mice

**Figure 8:**
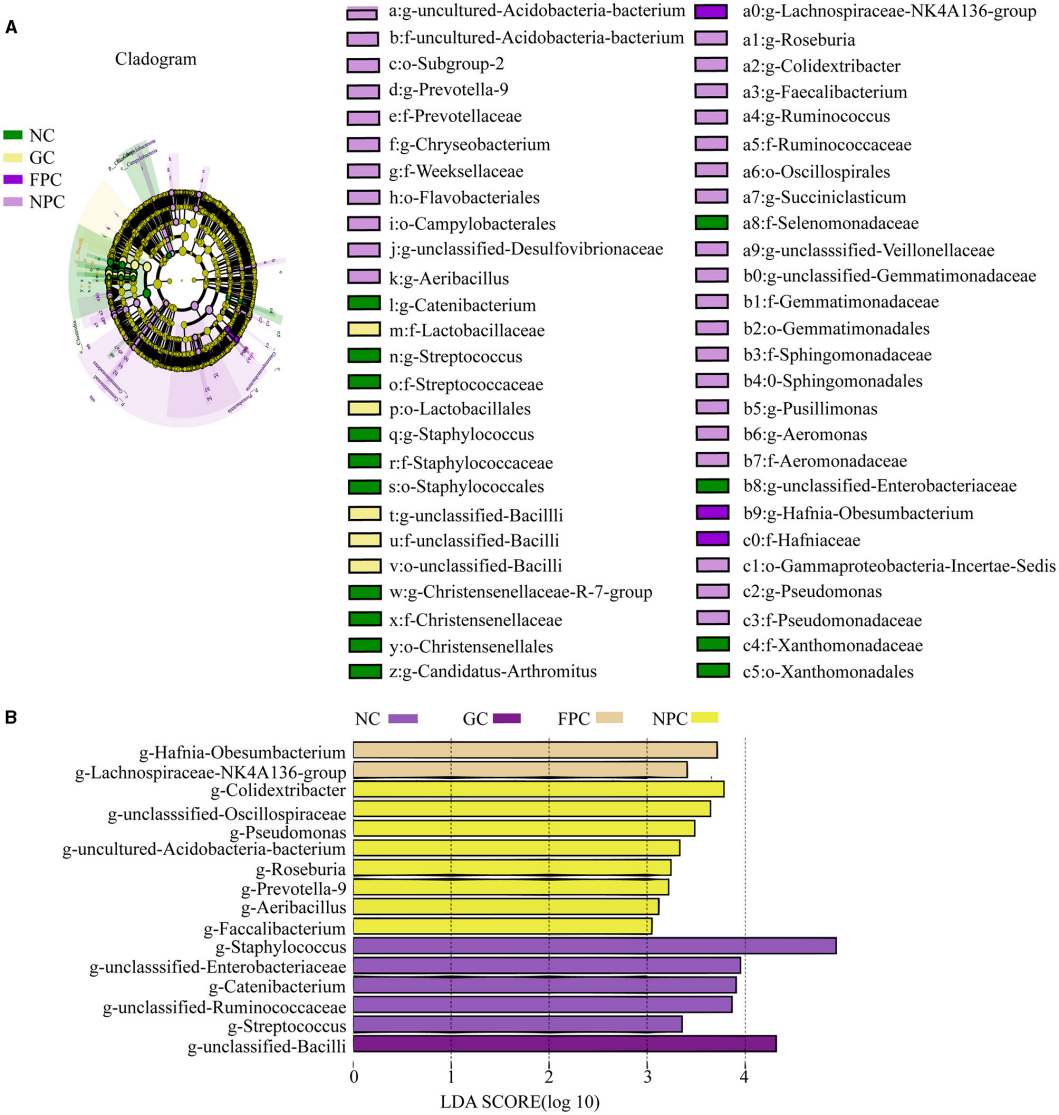
LDA SCORE analysis of the PFL group. **(A)** cladogram analysis; **(B)** LDA Score analysis. NC, normal control group; GC, general liquor group; FPC, fresh *Polygonatum* fermented liquor group; NPC, nine-steam-nine-bask *Polygonatum* fermented liquor group.

To further validate the regulatory effects of different liquors on the intestinal microbiota of mice, LEfSe analysis was conducted, with a linear discriminant analysis (LDA) score > 2 used as the screening criterion. Multiple comparisons were made among groups to identify characteristic genera with significant differences between groups. As shown in the Figures 8A, B, the NC, GC, FPC, and NPC groups included five, one, two, and eight characteristic genera, respectively. Among them, *Staphylococcus*, unclassified *Enterobacteriaceae*, and *Catenibacterium* were characteristic genera enriched in the NC group, unclassified *Bacilli* was the characteristic genus enriched in the GC group, *Lachnospiraceae* NK4A136 group and *Obesumbacterium* (*Hafnia*) were characteristic genera enriched in the FPC group, and *Colidextribacter*, unclassified *Oscillospiraceae*, and *Pseudomonas* were the significantly enriched characteristic genera in the NPC group.

### 3.10 Effects of PFL on the association between dominant genera in mice and environmental factors

**Figure 9:**
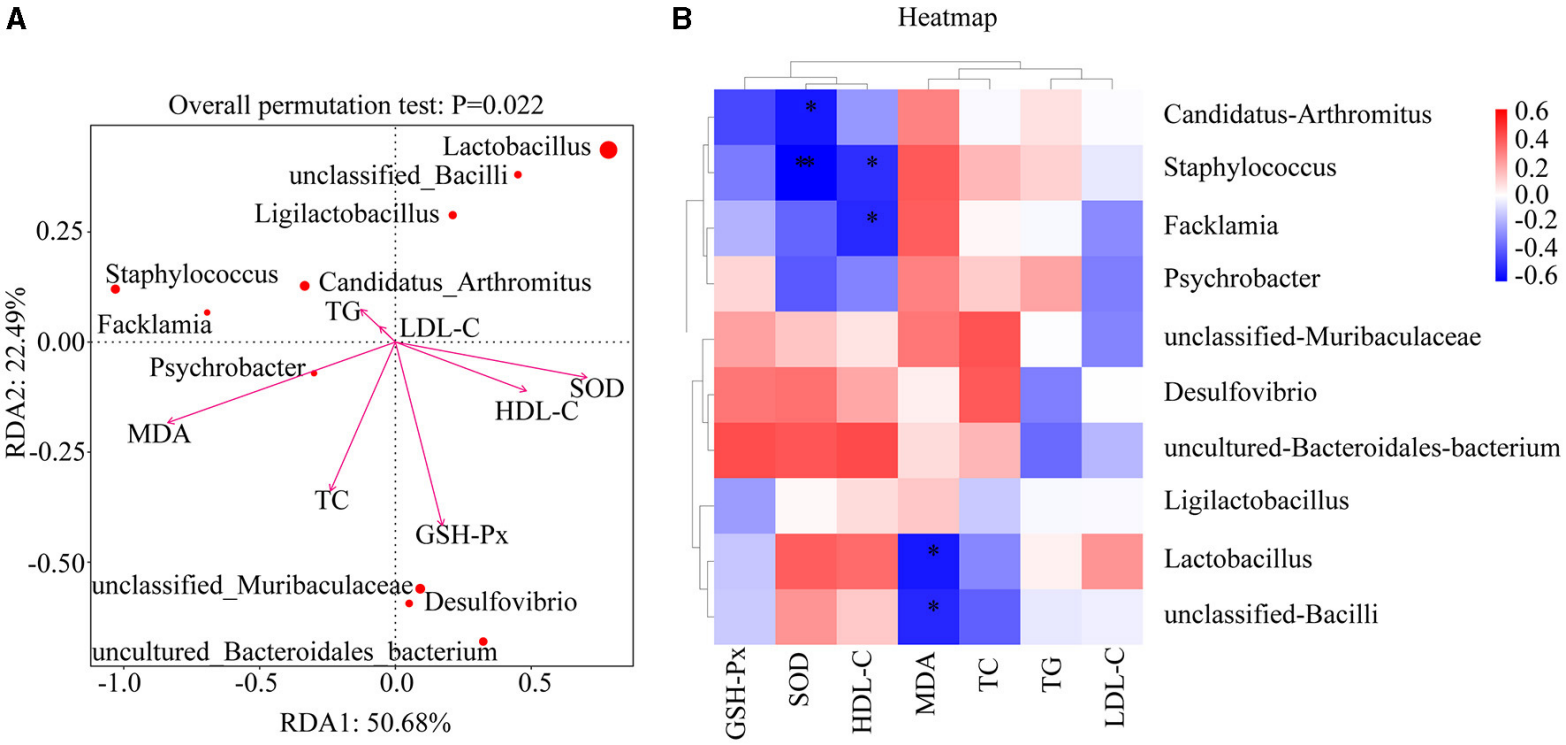
RDA correlation analysis of PFL. **(A)** RDA analysis between dominant microbiota and related indexes. **(B)** Heat map analysis. NC, normal control group; GC, general liquor group; FPC, fresh *Polygonatum* fermented liquor group; NPC, nine-steam-nine-bask *Polygonatum* fermented liquor group. *p < 0.05, **p < 0.01.

Using each relevant index as an environmental factor, RDA analysis was performed to analyze the correlation between the changes in the relative abundance of the top 10 dominant genera (with an abundance >1%) and environmental factors, and a heat map was used for accurate representation. As shown in Figure 9A, the length of the arrow represents the strength of the environmental factor's influence on community changes, with the arrows for GSH-Px, MDA, SOD, and TC being the longest, indicating their greatest impact. The closer the sample point is to the arrow, the stronger the impact of the environmental factor on the sample. When the sample is in the same direction as the arrow, it indicates a positive correlation between the environmental factor and the changes in the sample's species community. The illustration shows that GSH-Px has the strongest positive correlation with unclassified Muribaculaceae, *Desulfovibrio*, and uncultured Bacteroidales bacterium, MDA has the strongest positive correlation with *Psychrobacter*, and TC has the strongest positive correlation with unclassified Muribaculaceae, *Desulfovibrio*, uncultured Bacteroidales bacterium, and *Psychrobacter*. As shown in Figure 9B, MDA is significantly negatively correlated with *Lactobacillus* and unclassified *Bacilli* (*p* = 0.011, *p* < 0.05; *p* = 0.019, *p* < 0.05), SOD is significantly negatively correlated with *Staphylococcus* (*p* = 0.004, *p* < 0.01) and *Candidatus Arthromitus* (*p* = 0.012, *p* < 0.05), and HDL-C is significantly negatively correlated with *Staphylococcus* and *Facklamia* (*p* = 0.025, *p* < 0.05; *p* = 0.021, *p* < 0.05). In conclusion, there is a close association between each environmental factor and dominant genera in the intestine, indicating that PFL may improve lipid metabolism and oxidative stress by regulating the dominant genera in the intestine.

### 3.11 Effects of PFL on the metabolic function of mice

**Figure 10:**
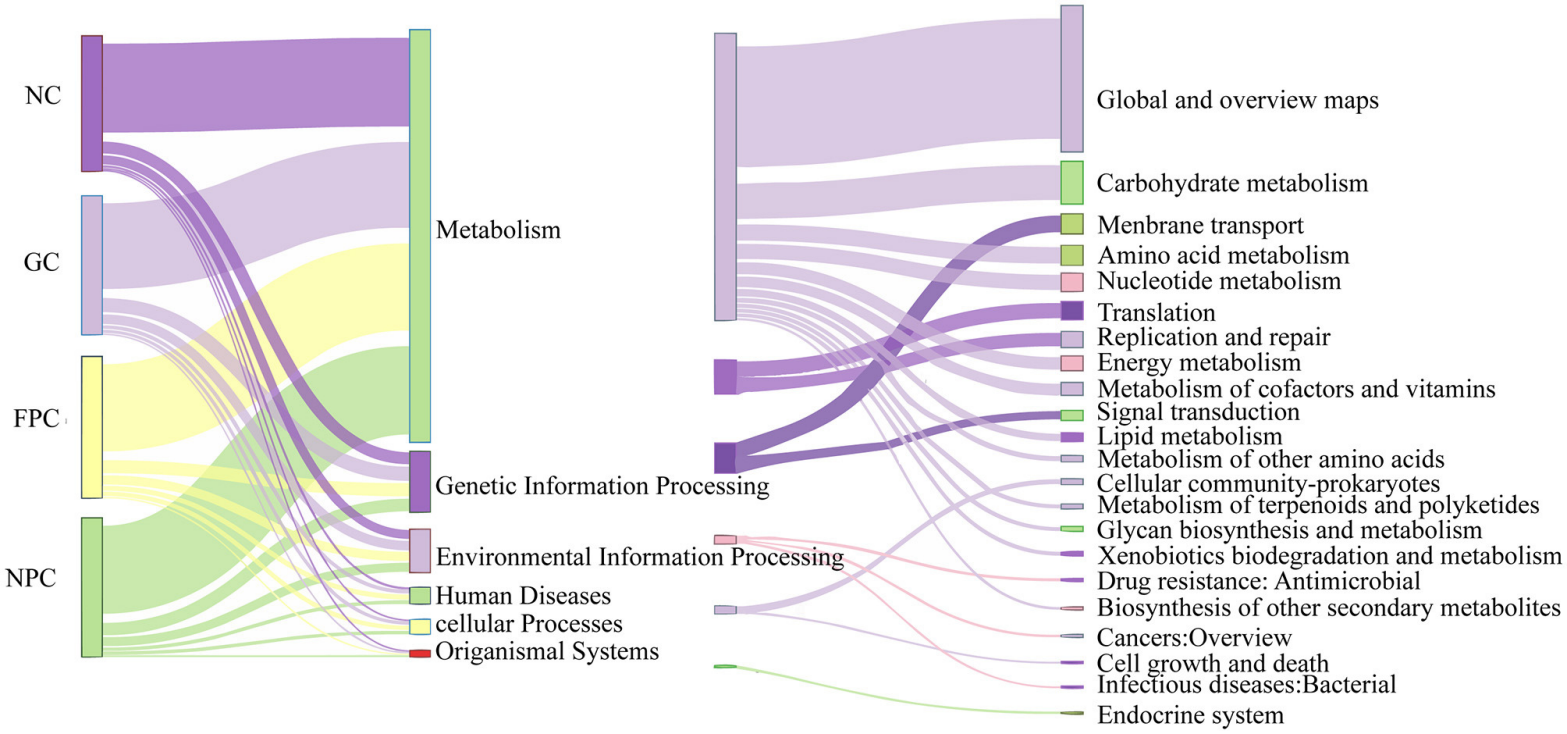
PFL KEGG functional analysis. NC, normal control group; GC, general liquor group; FPC, fresh *Polygonatum* fermented liquor group; NPC, nine-steam-nine-bask *Polygonatum* fermented liquor group.

To investigate whether changes in the composition of gut microbiota lead to functional changes, we predicted the functional profile of gut microbiota. As shown in Figure 10, the functions of gut microbiota were roughly divided into six categories, with 22 sub-functional classes at the second level. Among them, the sub-functional categories under “Metabolism” accounted for a relatively large abundance. They had a significant impact on the global and overview maps, carbohydrate metabolism, and membrane transport. Subsequently, we further analyzed the third-level sub-functional categories under “Metabolism” and found that metabolic pathways, biosynthesis of secondary metabolites, biosynthesis of antibiotics, and microbial metabolism in diverse environments had relatively large abundances, but there were no significant differences (*p* > 0.05).

## 4 Discussion

### 4.1 PFL may reduce the effect of sugar on body weight by influencing gut microbiota

In this study, it was observed that intragastric administration of regular liquor led to an increase in body weight in mice, while both types of PFL reversed this trend. Further investigation indicated that PFL could decrease the levels of TC, TG, and LDL-C in mice, while increasing the levels of HDL-C, suggesting a significant improvement in lipid metabolism. The liver, as the primary organ for lipid and lipoprotein synthesis and metabolism, showed a reduction in lipid droplet numbers in mice administered with PFL, as observed through HE staining. Combining macroscopic and microscopic indicators, it is speculated that PFL may have anti-obesity effects.

There is a reciprocal interaction between the liver and intestines through bile ducts, portal veins, and systemic circulation. Metabolites from the liver affect the gut microbiota and intestinal barrier function, while the gut microbiota also participate in regulating bile acid synthesis and lipid and sugar metabolism in the liver. To further explore the mechanism of action of PFL in reducing obesity, we analyzed the gut microbiota of mice from each group using 16S sequencing technology. An increase in the F/B ratio is considered a characteristic of obesity. Analysis of the correlation between dominant bacterial genera in mice and lipid metabolism indicators revealed a significant negative correlation between HDL-C and *Staphylococcus* and *Facklamia* (*p* = 0.025, *p* < 0.05; *p* = 0.021, *p* < 0.05). *Staphylococcus* is the most common pyogenic cocci and an important source of hospital-acquired infections. *Facklamia* is a gram-positive cocci with negative catalase activity and facultative anaerobic properties, considered an opportunistic pathogen.

Previous studies have shown that *Polygonatum* polysaccharides can regulate the relative abundance and diversity of the gut microbiota, promote the restoration of the intestinal barrier, inhibit the entry of lipopolysaccharides into the circulation, alleviate inflammatory responses, and ultimately prevent lipid metabolism disorders (31). Therefore, it is speculated that PFL may reduce the abundance of pathogenic bacteria such as *Staphylococcus* and *Facklamia*, enhance intestinal barrier function, and thus play a role in lowering lipid levels.

### 4.2 PFL may alleviate oxidative stress by influencing gut microbiota

Previous studies have shown that alcohol can cause oxidative stress mediated by intracellular reactive oxygen species (32). The liver is the organ most severely affected by oxidative stress, as excessive reactive oxygen species can lead to DNA damage, mitochondrial dysfunction, and lipid peroxidation in liver cells. This study found that PFL could increase the levels of SOD and GSH-Px in mouse livers while reducing the levels of MDA. In particular, NPFL significantly increased the levels of GSH-Px in mouse livers (*p* = 0.022, *p* < 0.05). Therefore, PFL is considered to have an antagonistic effect on alcohol and can alleviate oxidative stress and protect the liver (33–35).

To further investigate the mechanism by which PFL antagonizes alcohol, we conducted a correlation analysis between dominant bacterial genera in mice and oxidative stress indicators. The results showed a significant negative correlation between MDA and *Lactobacillus* and unclassified *Bacilli* (*p* = 0.011, *p* < 0.05; *p* = 0.019, *p* < 0.05), and between SOD and *Staphylococcus* (*p* = 0.004, *p* < 0.01) and *Candidatus Arthromitus* (*p* = 0.012, *p* < 0.05). Multiple studies have shown that most *Lactobacillus* and *Bacillus* genera have strong antioxidant activity, while most *Staphylococcus* significantly increases oxidative stress levels in the body (36–41). *Candidatus Arthromitus* is a major member of the Deferribacteres phylum and can cause various infections, intestinal diseases, and inflammation. Therefore, PFL is considered to primarily reduce oxidative stress and protect the liver by upregulating *Lactobacillus* and unclassified *Bacilli* while downregulating the abundance of *Staphylococcus* and *Candidatus Arthromitus*.

### 4.3 PFL may improve brain neurological function through the brain–gut axis

Alcohol can affect various neurotransmitter receptors, thereby disrupting neuronal function (42). At non-lethal doses, alcohol primarily damages brain function by binding to GABA receptors in the brain. BDNF plays an important role in the development, learning, and memory of the central nervous system (43). Previous studies have shown that BDNF, as an alcohol-dependent regulatory system in the human brain, can effectively block alcohol intake (44). The results of this study showed that the levels of GABA and BDNF in mouse brain tissue increased in the group administered NPC, indicating a good protective effect of this liquor on brain tissue.

PFL may exert a protective effect on brain tissue through the brain–gut axis. The complex bidirectional regulatory relationship between the gut microbiota and the traditional brain–gut axis is referred to as the “brain–gut axis,” which is a bidirectional regulatory pathway connecting the central nervous system with gastrointestinal digestive functions (45). *Polygonatum* plays a role in improving the neurological function of the brain. Studies have shown that *Polygonatum* polysaccharides regulate the BDNF pathway through the gut microbiota and increase BDNF levels (23). In addition, *Polygonatum* polysaccharides also promote the proliferation of bacteria that produce GABA, increasing GABA levels in the body (46). The gut microbiota influences neural function in the brain through the brain–gut axis. Gut probiotics have the capacity to directly generate neurotransmitters, such as *Bacteroides* and lactic acid bacteria that produce GABA. Furthermore, gut probiotics can also regulate pro-BDNF to BDNF conversion and elevate BDNF levels in the hippocampus. This study suggests that consuming PFL may significantly increase beneficial bacteria like *Bacteroides* and *Lactobacillus* genus. Therefore, PFL may improve neurological function of the brain by regulating the structure of gut microbiota, increasing the proportion of probiotics, and promoting the increase of intracranial GABA and BDNF levels.

## 5 Conclusion

This study suggests that functional PFL has a positive impact on lipid levels and antioxidants in normal mice. These effects are potentially achieved by influencing the composition and diversity of intestinal microbiota, promoting increased levels of GABA and BDNF in the brain. Nonetheless, further longitudinal investigations are necessary to explore the microecological mechanisms by which specific gut bacterial genera affect the gut–brain axis and to ascertain the precise mechanistic pathways that underlie the functional PFL. Similar studies have always highlighted the medicinal value of PFL, but this study focused on the general population to explore the possibility of PFL as a functional food for long-term consumption, aiming to emphasize the homology of *Polygonatum* medicine and food (47). In conclusion, this study revealed the effect of fermentation on *Polygonatum* and its effect on the brain–gut axis in the mouse model, which points to a significance for the general population to achieve lipid-lowering and antioxidant effects through long-term drinking of fermented *Polygonatum* liquor.

## Data availability statement

The datasets presented in this study can be found in online repositories. The names of the repository/repositories and accession number(s) can be found at: https://www.ncbi.nlm.nih.gov/bioproject/PRJNA1056474/.

## Ethics statement

The animal study was approved by the Animal Ethics and Welfare Committee of Hunan University of Chinese Medicine. The study was conducted in accordance with the local legislation and institutional requirements.

## Author contributions

XY: Writing – original draft. LF: Writing – original draft. JS: Writing – original draft. ZT: Writing – review & editing. WZ: Writing – original draft. MP: Writing – review & editing. NX: Writing – review & editing.

## Footnotes

^1^China Pharmacopoeia 2020 edition. One [S].319.
